# Design and Analysis of a Microgripper with Three-Stage Amplification Mechanism for Micromanipulation

**DOI:** 10.3390/mi13030366

**Published:** 2022-02-25

**Authors:** Yuan Hong, Yimin Wu, Shichao Jin, Dayong Liu, Baihong Chi

**Affiliations:** Space Star Technology Co., Ltd., Beijing 100086, China; wuym503@163.com (Y.W.); shichao182@163.com (S.J.); liudywork@sina.com (D.L.); baihong1988@163.com (B.C.)

**Keywords:** microgripper, mechanism design, bridge type mechanism, micromanipulation

## Abstract

This paper proposes a novel microgripper with two working modes. The microgripper is designed with symmetric structure and each part is actuated by one piezoelectric actuator, respectively. To achieve desired output displacement, each part of the microgripper is designed with three-stage amplification mechanism to amplify the displacement of the PZT actuator. According to the size of the microobjects, the grasping operation can be completed by one finger moving or two fingers moving simultaneously. Then, the theoretical analysis is carried out to calculated the key characteristics, including amplification, input stiffness and frequency. Finite element analysis (FEA) is conducted to optimize the structural parameters and investigate the performance of the microgripper. Finally, a prototype is machined by wire electro-discharge machining (WEDM) method and experiments are carried out to verify the performance of the microgripper. The results indicate that the amplification is 10.41 and the motion stroke of one jaw is 118.34 µm when the input voltage is 100 V. The first natural frequency is 746.56 Hz. By picking and placing the wires with different diameters and slices with different thickness, the grasping stability is verified.

## 1. Introduction

In recent decades, research in the microscopic fields such as biological engineering [1,2], microelectronics industrial [3,4], and precise assembly [5,6], have attracted more and more attention. To achieve precise and stable operation of the microobjects, it is crucial to develop series of microgripper with good performance.

Different kinds of microgrippers have been developed to fulfill the requirements of different targets. According to the difference among actuators, the microgripper can be divided into electrothermal [7,8], electrostatic [9,10] and piezoelectric (PZT) [11,12] actuating microgrippers. Regarding electrothermal microgripper, it can generate a large displacement and force under a low excitation voltage and the compact structure is another advantage of this kind of microgripper. But the drawbacks of high operation temperature, nonlinear movement and low sensitivity limit their application. Due to the compact structure, the electrostatic microgrippers are usually used to manipulate the objects with the size of tens of microns. However, the grasping produced by the electrostatic microgrippers are usually small, which influence the grasping stability. The PZT actuating microgripper possess the advantages of high force output to weight ratio, fast response and zero backlash, which has been widely used in micromanipulation [13]. As mentioned above, the features of different kind of microgrippers can be summarized in Table 1.

To achieve large amplification ratio, most of the existing microgrippers are designed with symmetric structure and two fingers are driven by one actuator. A high compliant microgripper with symmetric structure was developed and each part was designed with a two-stage lever mechanism [14]. The experimental results indicates that the microobjects with the scale ranging from hundreds of microns to a few can be operated by this microgripper. A novel microgripper driven by one PZT actuator was proposed in reference [15], which can achieve the maximum jaw displacement of 134 μm and a high amplification ratio of 15.5. Scott Russell mechanism and lever mechanism were employed to construct the amplification mechanism to amplify the input displacement. Liang et al. [16] proposed a piezoelectric actuated microgripper, which can achieve an amplification ratio of 22.8 and an output displacement of 190 μm corresponding to the 100 V input voltage. A high-power piezo-driven microgripper based on the principle of combined amplification was proposed in [17]. The actual magnification was 23.2 and different shapes of microgripper can be manipulated by the microgripper. A combination of flexure hinges and bias spring mechanism with appropriate mechanical transmission were utilized to develop a microgripper which can achieve a maximum stroke of 100 μm [18]. Qian et al. [19] proposed a microgripper with three-stage amplification mechanism. A new position/force model used to describe both kineto-statics and dynamics of the microgripper is established. Another two-finger microgripper with three-stage amplification mechanism was developed in [20], where three materials were adopted to carry out the model analysis. However, two fingers of the abovementioned microgrippers are actuated by one PZT actuator and move simultaneously. The inevitable error during fabrication and assembly may result in asymmetric motion of grasping jaws. 

In addition, some researchers developed a kind of microgripper with just one movable finger. Liang et al. [21] designed an asymmetrical microgripper with one movable finger. Theoretical analysis and finite element analysis (FEA) were adopted to investigate the characteristics of the microgripper, and the results shown that the amplification ratio can reach 16.78. A compliant constant-force microgripper with one movable finger was designed in reference [22]. The maximum output displacement of 220 μm can be achieved. Das et al. [23] suggested a PZT actuated microgripper with a bridge-type mechanism and a lever mechanism to amplify the output of the PZT actuator. The microgripper can achieve an output displacement of 483.31 μm with a displacement amplification ratio of 15.5. Compared with the microgripper with symmetric structure, the compact structure needs to be sacrificed to obtain desired grasping ranges. The comparisons between these related other works are summarized in Table 2.

This paper proposes a compliant microgripper with three-stage amplification mechanism and each part is actuated by one PZT actuator. Therefore, the grasping operation by one finger movement or two finger movement can be determined according to the size of the microobjects. When the grasping mode of two finger movement is adopted to pick the tiny object, the output displacement of two fingers can be controlled to be consist by the displacement controller, which can avoid the disadvantages of the microgrippers driven by one PZT actuator. The mechanism design process is described in Section 2. Theoretical analysis, FEA and experimental tests are carried out to investigated the characteristics of the developed microgripper.

## 2. Mechanism Design of the Microgripper

**Figure 1:**
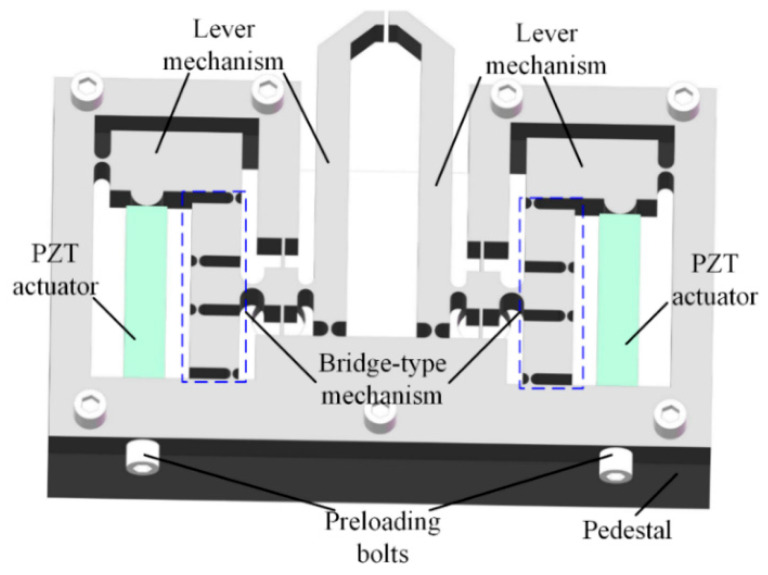
The CAD drawing of the proposed microgripper.

In this paper, a compliant microgripper with symmetric structure driven by two PZTs is developed and the prototype of the microgripper generated in Solidworks is as shown in Figure 1. The microgripper consists of two fingers, two PZT actuators two preload bolts and two three-stage compliant amplification mechanisms. The microgripper is installed on a pedestal to eliminate the influence of the friction force.

The microgripper is designed as symmetric structure which can be divided into left part and right part, where each part is actuated by one actuator, respectively. Hence, According to the size of the gripped objects, one finger moving grasping mode or two finger moving grasping mode can be adopted to grip the object. When the grasping mode of two finger movement is adopted to pick the tiny object up, the output displacement of two fingers can be controlled to be consist by the displacement controller, which can avoid the disadvantages of the microgrippers driven by one PZT actuator.

The output displacement of the PZT actuator is small which can not meet the requirements of large stroke. To enlarge the output displacement of the fingers, a three-stage amplification mechanism including two lever magnification mechanisms and a bridge-type displacement amplification mechanism is employed at each part. As shown in Figure 1, the PZT actuators are installed inside the bridge-type mechanism and the moving direction of the PZT actuator is along vertical axis, while the desired motion of the finger needs to be along horizontal axis. Hence, a reversing mechanism needs to be designed to obtain the desired transmission direction of displacement. The bridge-type mechanism not only possess the function of amplifying the output displacement but also can change the transfer direction of the movement [24,25], so that the desired motion of the two fingers can be achieved to complete grasping operation. Between the output terminal of the bridge-type mechanism and the base, two leaf-spring flexure hinges (LSFHs) are equipped to ensure the motion transmitting direction to reduce the parasitic displacement. To further amplify the output displacement, another leverage mechanism is adopted to connect with the output end of the bridge-type mechanism.

Right circular flexure hinges (RCFHs) are more precise than other flexible hinges in keeping the rotational center position, so the bridge-type mechanism and lever mechanism are designed with RCFHs.

## 3. Modeling and Characteristics Analysis

The characteristics of the microgripper such as amplification ratio, input stiffness and natural frequency are analyzed by theoretical calculation. Based on the pseudo rigid body model (PRBM) method [26] and the matrix-based compliance modeling (MCM) method [27], the relationships between the amplification ratio, the input stiffness and the key parameters of the microgripper are established, respectively. This section may be divided by subheadings. It should provide a concise and precise description of the experimental results, their interpretation, as well as the experimental conclusions that can be drawn.

### 3.1. Amplification Ratio of the Microgripper

**Figure 2:**
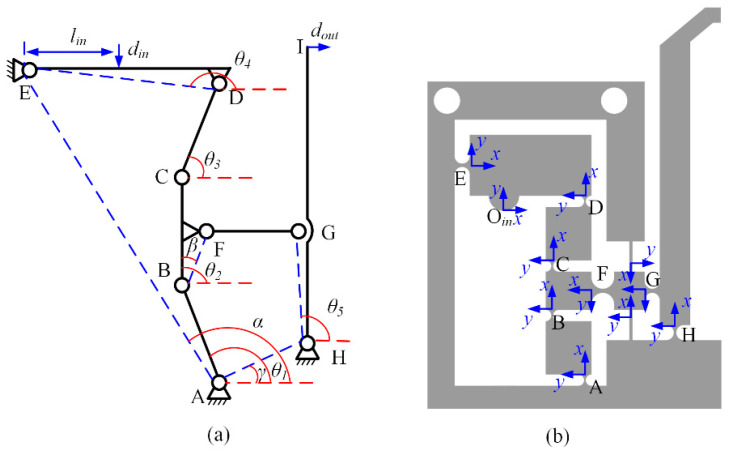
(**a**) The simplified structure of left part using PRB model, (**b**) the schematic diagram of left part.

Due to left-right symmetry of the microgripper, the left part is selected to be analyzed and can be simplified as shown in Figure 2a according to the PRBM method. It can be seen from Figure 2a, the RCFHs are equivalent as rotational hinges and the linkages of A-B-C-D-E-F-G-H can be considered as six-bar mechanism. *i* (*i* = *A*, *B*, *C*, *D*, *E*, *F*, *G*, *H*) are the rotational center and *l_j_* (*j* = *AB*, *BC*, *CD*, *ED*, *FG*, *HG*, *AH*, *AE*) are the length of these linkages. *φ_i_* (*i* = 1, 2, …, 5) are the initial angular of the linkages *AB*, *BC*, *CD*, *HG*. According to the geometric characteristics of the six-bar mechanism, the following relationships can be obtained [28]

(1) $$\left\{\begin{array}{c} l_{AB}e^{i\theta_{1}}+l_{BC}e^{i\theta_{2}}+l_{CD}e^{i\theta_{3}}=l_{AE}e^{i\alpha }+l_{ED}e^{i\theta_{4}}\\ l_{AB}e^{i\theta_{1}}+l_{BF}e^{i\left(\theta_{2}-\beta \right)}+l_{FG}=l_{AH}e^{i\gamma }+l_{Hg}e^{i\theta_{5}} \end{array}\right.$$

where *α*, *β*, *γ* are the angular position of linkages *AE*, *AH* and *BF*.

Substituting the Euler equation of complex variable function *e^iθ^* = cos*θ* + *i*sin*θ* into Equation (1), the following two equations can be derived

(2) $$\left\{\begin{array}{l} l_{AB}\cos\theta_{1}+l_{BC}\cos\theta_{2}+l_{CD}\omega_{3}\cos\theta_{3}=l_{AE}\cos\alpha +l_{ED}\omega_{4}\sin\theta_{4}\\ l_{AB}\sin\theta_{1}+l_{BC}\sin\theta_{2}+l_{CD}\omega_{3}\sin\theta_{3}=l_{AE}\sin\alpha +l_{ED}\omega_{4}\sin\theta_{4} \end{array}\right.$$

(3) $$\left\{\begin{array}{l} l_{AB}\cos\theta_{1}+l_{BF}\cos\left(\theta_{2}-\beta \right)\;=l_{AH}\cos\gamma +l_{HG}\cos\theta_{5}\\ l_{AB}\sin\theta_{1}+l_{BF}\sin\left(\theta_{2}-\beta \right)\;=l_{AH}\sin\gamma +l_{HG}\sin\theta_{5} \end{array}\right.$$

Differentiating the equations with respect to time, the following two equations can be obtained

(4) $$\left\{\begin{array}{c} l_{AB}\omega_{1}\sin\theta_{1}+l_{BC}\omega_{2}\sin\theta_{2}+l_{CD}\omega_{3}\sin\theta_{3}=l_{ED}\omega_{4}\sin\theta_{4}\\ l_{AB}\omega_{1}\cos\theta_{1}+l_{BC}\omega_{2}\cos\theta_{2}+l_{CD}\omega_{3}\cos\theta_{3}=l_{ED}\omega_{4}\cos\theta_{4} \end{array}\right.$$

(5) $$\left\{\begin{array}{c} l_{AB}\omega_{1}\sin\theta_{1}+l_{BF}\omega_{2}\sin\left(\theta_{2}-\beta \right)\;=l_{HG}\omega_{5}\sin\theta_{5}\\ l_{AB}\omega_{1}\cos\theta_{1}+l_{BF}\omega_{2}\cos\left(\theta_{2}-\beta \right)\;=l_{HG}\omega_{5}\cos\theta_{5} \end{array}\right.$$

According to Equations (4) and (5), the angular velocity of *ω*_2_, *ω*_4_ and *ω*_5_ can be calculated as

(6) $$\left\{\begin{array}{l} \omega_{2}=a\omega_{1}=\frac{l_{AB}\sin\left(\theta_{5}-\theta_{1}\right)}{l_{BF}\sin\left(\theta_{2}-\beta -\theta_{5}\right)}\omega_{1}\\ \omega_{4}=b\omega_{1}=\frac{l_{AB}\sin\left(\theta_{3}-\theta_{1}\right)\;+\;al_{AC}\sin\left(\theta_{3}-\theta_{2}\right)}{l_{ED}\sin\left(\theta_{3}-\theta_{4}\right)}\omega_{1}\\ \omega_{5}=\frac{l_{AB}\sin\left(\theta_{2}-\beta -\theta_{1}\right)}{l_{HG}\sin\left(\theta_{2}-\beta -\theta_{5}\right)}\omega_{1} \end{array}\right.$$

The displacement magnification ratio is defined as the ratio of the output displacement to the input displacement. Hence, based on the simplified model shown in Figure 2, the amplification ratio for each part can be written as

(7) $$\lambda =\frac{d_{out}}{d_{in}}=\frac{\omega_{5}l_{HI}}{\omega_{4}l_{ED}}$$

### 3.2. The Natural Frequency of the Microgripper

Firstly, the input stiffness is calculated based on MCM method to establish the relationship between input force and deformation. According to reference [29], the 3 × 3 compliant matrix of RCGHs and LSFHs can be obtained. Then, the stiffness of the input end can be calculated according to the series-parallel relationships of the flexible hinges as shown in Figure 2b.

It can be seen from Figure 2b, the flexible hinge *G* and flexible hinge H are connected in series, and then connected in parallel with two LSFHs. Hence, the compliance *C*_1_ of these hinges with respect to input point *O_in_* can be written as

(8) $$C_{1}={\left[{\left(C_{H}+C_{G}\right)}^{\prime }+C_{P1}^{\prime }+C_{P2}^{\prime }\right]}^{\prime }+C_{F}$$

where $C_{P1}^{\prime }$ and $C_{P2}^{\prime }$ are the compliance of two LSFHs.

Flexible hinges *A* and *B* are connected in serial with respect to *O_in_*

(9) $$C_{2}=C_{A}+C_{B}$$

Similarly, the compliance of flexible hinges *C* and *D* with respect to *O_in_* can be calculated as

(10) $$C_{3}=C_{C}+C_{D}$$

Then, the input compliance of the microgripper can be derived as

(11) $$C_{in}={\left[{\left({\left(C_{{}_{1}}^{\prime }+C_{2}^{\prime }\right)}^{\prime }+C_{3}\right)}^{\prime }+C_{E}^{\prime }\right]}^{\prime }$$

Hence, the input stiffness can be written as

(12) $$k_{in}=C_{{}_{in}}^{\prime }\left(2,2\right)$$

Next, Lagrange’s equation is employed for the dynamic modeling of the microgripper.

(13) $$\frac{d}{dt}\left(\frac{\partial T}{\partial \dot{q_{i}}}\right)-\frac{\partial T}{\partial q_{i}}+\frac{\partial V}{\partial q_{i}}=F$$

where *T* represents the total kinetic energy of the system, *V* represents the total potential energy of the system, and *q_i_* is the generalized coordinate of the system, $\dot{q_{i}}$ is the generalized speed of the system, and *F_i_* is the generalized force corresponding to *q_i_*.

The kinetic energy of the entire system can be calculated as

(14) $$T=\frac{1}{2}\left[J_{ED}\omega_{4}^{2}+J_{CD}\omega_{3}^{2}+J_{AB}\omega_{1}^{2}+J_{HI}\omega_{5}^{2}+\left(m_{BC}+m_{FG}\right)v_{BC}^{2}\right]$$

where *J_ED_*, *J_CD_*, *J_AB_*, *J_HI_* represent the rotational moment of the corresponding linkages *ED*, *CD*, *AB*, *HI*. *m_BC_*, *m_FG_* denote the mass of the linkages of *BC* and *FG*.

The potential energy of the entire system is expressed as

(15) $$V=\frac{1}{2}k_{in}d_{in}^{2}$$

The dynamic equation of the microgripper can be written as

(16) $$M{\overset{..}{d}}_{in}+k_{in}d_{in}=0$$

where *M* is the equivalent mass.

Then, the natural frequency in Hz can be derived as

(17) $$f=\frac{1}{2\pi }\sqrt{\frac{k_{in}}{M}}$$

Then, the key parameters of the microgripper are optimized to obtained the desired amplification ratio and high bandwidth, during which the Equations (7) and (17) are selected as objective function. And the key parameters are optimized as: *r_A_* = *r_B_* = *r_C_* = *r_D_* = 0.75 mm, *t_A_* = *t_B_* = *t_C_* = *t_D_* = 0.2 mm, *r_E_* = *r_H_* = *r_G_* = 1 mm, *r_F_* = 1.5 mm, *t_H_* = 0.3 mm, *t_E_* = 0.4 mm, *t_F_* = *t_G_* = 0.5 mm, where *r_i_* (*i*= *A*, *B*…*G*, *H*) denote the radius of the RCGHs, *t_i_* (*i* = *A*, *B*…*G*, *H*) are the thickness of the RCGHs. Substituting the key parameters into Equations (7) and (17), the amplification ratio and the natural frequency can be calculated as 12.5 and 707.83 Hz.

## 4. Finite Element Analysis

In order to verify the theoretical model of the designed microgripper, commercial software ABAQUS is used for finite element analysis. The material of the microgripper is designed as Al-7075 and the material parameters are set as shown in Table 3. During simulation analysis, zero displacements are assigned on the surfaces of the fixing holes to immobilize the mechanism and the input displacement is applied on the input terminal of the microgripper for the static analysis.

**Figure 3:**
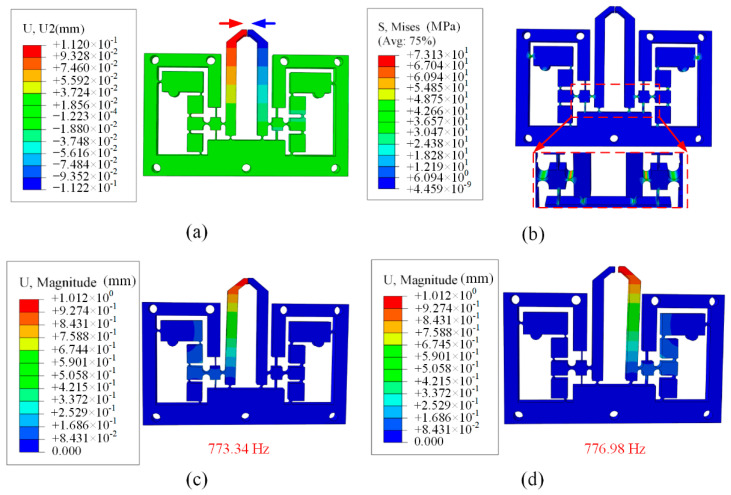
The FEA results of (**a**) the deformation behavior, (**b**) stress distribution, (**c**) the first modal shapes and (**d**) the second modal shapes.

As is shown in Figure 3a, when applied a displacement with the amplitude of 10 μm to the input terminals, the maximum output displacement of the microgripper at the output end is 133.2 μm. The displacement amplification ratio can be calculated as 11.2 for each part, and the maximum amplification can be calculated as 22.4 when grasping mode of two finger movement is adopted. Figure 3b suggests that the maximum stress of the microgripper is 73.13 Mpa corresponding to the input displacement of 10 μm, much less than the yield stress of the material 503 Mpa.

Besides, it is necessary to carry out the modal analysis to investigate the dynamic performance of the microgripper. For the free vibration, the first mode shapes of two part of the microgripper are along the grasping direction and the corresponding frequencies are 773.34 Hz and 776.98 Hz, respectively, as shown in Figure 3c,d. The results indicate that the developed microgripper can pick and place the objects with high speed.

## 5. Experimental Tests

**Figure 4:**
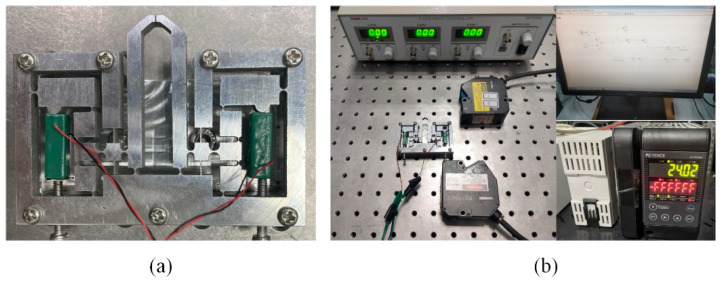
(**a**) The prototype of the proposed microgripper, (**b**) the experimental setup of the microgripper testing system.

This section is not mandatory but can be added to the manuscript if the discussion is unusually long or complex. The material of Al-7075 and the wire electro-discharge machining (WEDM) method are used to fabricate the prototype of the proposed microgripper as shown in Figure 4, where two PZT actuators (type: PSt150/5*5/20 L 5 × 5/18) are installed between the input end and the base by two preloading bolts. And the dimension of the microgripper is designed with 80 mm × 56 mm × 5 mm. Figure 4 also shows other experimental setup. The laser displacement sensors are adopted to measure the output displacement of the finger. A voltage amplifier is used to amplify the voltage required for the PZT actuators. The strain gauge glued on the surface of the jaw is used to measure the grasping force. The controlling signals generated by a computer are transferred to PZT actuator by a D/A board. And the signals collected by the sensors are read simultaneously by the computer through a A/D board.

### 5.1. Open-Loop Test

**Figure 5:**
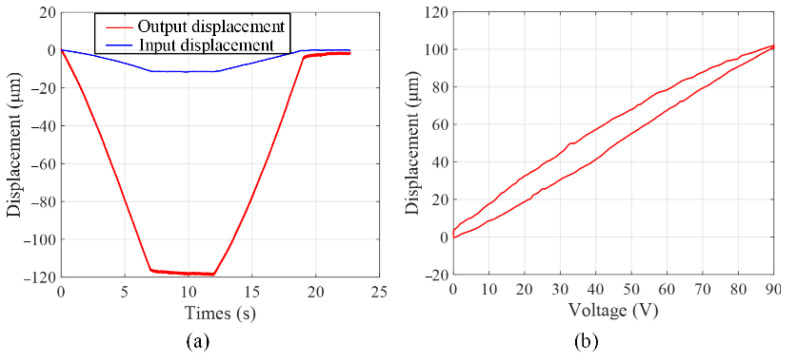
Open-loop tests of (**a**) amplification ratio test, (**b**) hysteresis nonlinearity test.

Firstly, the amplification ratio was tested by applying a trapezoid voltage signal with the amplitude of 100 V on the PZT actuator. The output displacements at finger and the input end are collected by two laser displacement sensors. The testing results are summarized in Figure 5a. It is can be seen from Figure 5a the amplification ratio of one part is 10.41 and the maximum output displacement is 118.46 μm corresponding to the voltage with the amplitude of 100 V. The displacement amplification ratio can be calculated as 20.7 when grasping mode of two finger movement is adopted.

Figure 5b shows the inherent characteristic of hysteresis nonlinearity introduced by PZT actuator. As can be seen from Figure 5b that when the voltage rises and falls, the same voltage corresponds to two different output displacements, which will affect operation precision and stability.

**Figure 6:**
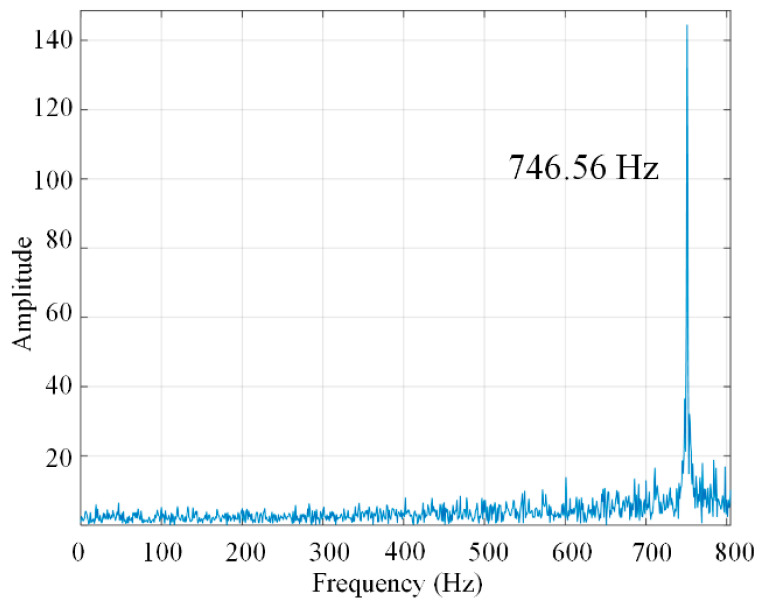
The experimental result of the frequency test.

Next, the frequency response of the gripper is generated by a swept-sine approach. And a swept sine signal with amplitude of 10 V and a frequency from 0.001 Hz to 1000 Hz is applied on the PZT actuator. The transfer function is shown in Figure 6. The result shows that the working vibration modes are excited and the corresponding frequencies are 746.56 Hz for the first natural frequency. The working vibration modes’ frequencies are slightly smaller than the results by the FEA. The fabricating error and the residual stress of the microgripper may have a great influence on it.

**Figure 7:**
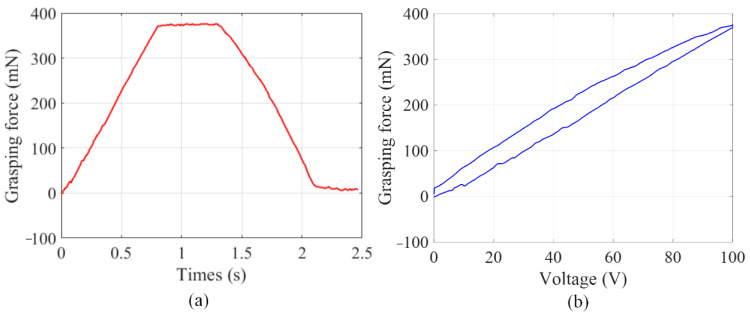
The grasping force test, (**a**) trapezoid response, (**b**) hysteresis nonlinearity.

Then, experiments of copper wire with the diameter of 0.5 mm grasping were carried out to estimate the grasping force, and the results were summarized as shown in Figure 7. When the input voltage applied on the PZT actuators are 100 V, the grasping force can reach 366.7 mN.

### 5.2. Close-Loop Tests

To improve the performance and eliminate the hysteresis nonlinearity, a position PID controller is introduced to obtain the desired output displacement. The PID parameters are tuned by series of experiments, and the values are set as: *K_P_* = 5.6, *K_I_* = 0.23, *K_D_* = 0.0001.

**Figure 8:**
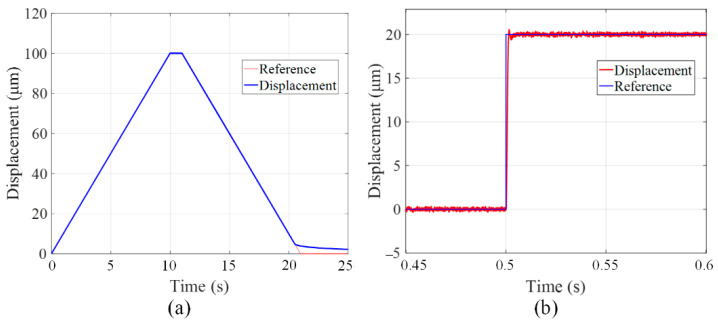
(**a**) The trapezoid response and (**b**) step response of the microgripper under close-loop control.

In order to verify the hysteresis characteristics under closed-loop conditions, a trapezoid displacement signal is applied on the microgripper. The output displacement is plotted in Figure 8a. As can be seen from the experimental results, hysteresis nonlinearity has been compensated significantly. Then, A step response test is carried out to investigate the accuracy of the microgripper. When a step displacement signal with the amplitude of 20 μm is applied on the PZT actuator, the actual output displacement at finger is collected by the laser displacement sensor and summarized in Figure 8b. According to the experimental data, the overshoot and the settle time of the step response signal are 2.75% and 39 ms, respectively.

**Figure 9:**
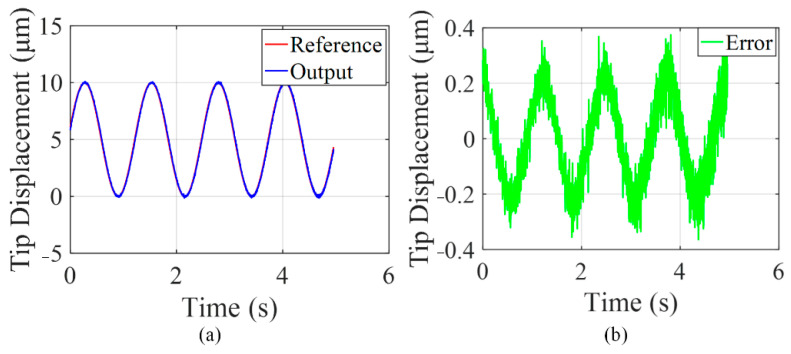
Position tracking experimental results of the microgripper: (**a**) the tracking trajectory, (**b**) the position tracking error.

Then, a trajectory tracking experiment by applying a sine wave signal on the PZT actuator is provided. The motion trajectory of the finger is collected by the laser displacement sensor and illustrated in Figure 9a. The difference between reference signal and actual trajectory is summarized as shown in Figure 9b, where the maximum position tracking error reach to 0.3 μm corresponding to the amplitude of 10 μm. The tracking percentage is obtained as 3% of the corresponding desired output displacement.

**Figure 10:**
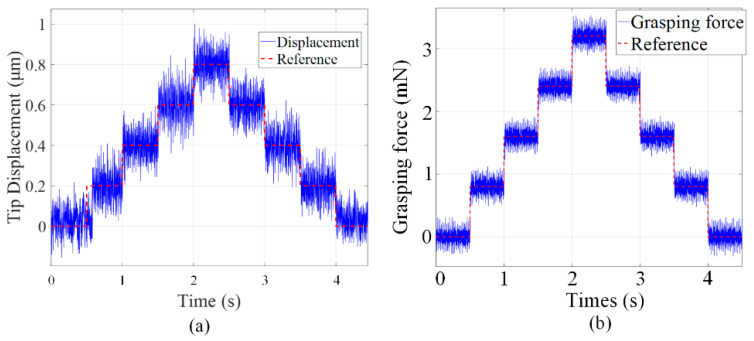
(**a**) The motion resolution and (**b**) the grasping force resolution, of the microgripper.

Finally, the displacement resolution is tested by applying a stairway-type signal on the PZT actuator. The constant time and the amplitude for each step of the stairway-type signal are set as 0.5 s and 0.2 μm, respectively. The position resolution is tested and the output displacement at finger is illustrated in Figure 10a. The experimental results indicated that the position resolution is 0.2 μm. Similarly, the grasping force resolution is tested and the results is summarized in Figure 10b. It is shown that the grasping force resolution can reach 0.8 mN.

### 5.3. Grasping Ability Test

**Figure 11:**
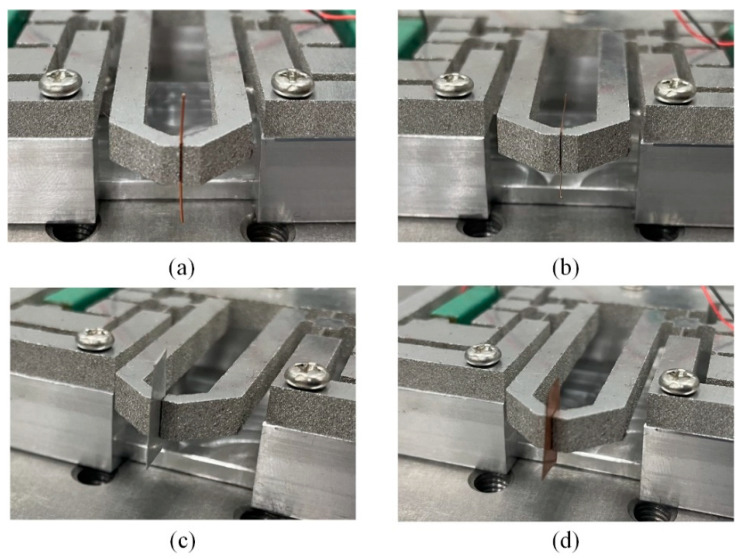
The manipulation of the: (**a**) wire with diameter of 200 μm, (**b**) wire with diameter of 100 μm, (**c**) slice with the thickness of 200 μm, (**d**) slice with the thickness of 150 μm.

The grasping stability of the microgripper is tested by grasping-holding-releasing the metal wire with different diameters and the slices with different thickness. The grasping process is captured by camera as shown in Figure 11. The initial gap between two fingers is set as 500 μm, and it can be adjusted by two preloading bolts. Figure 11a shows a wire with the diameter of 200 μm and the weight of 2.25 mg is grasped stably by the microgripper with the grasping mode of one finger movement. Similarly, the slice with the thickness of 200 μm and the weight of 9 mg is manipulated by the proposed microgripper, as shown in Figure 11c. To achieve larger grasping displacement, the grasping mode where two fingers move simultaneously is selected to pick a wire with the diameter of 100 μm and the weight of 0.56 mg. It can be seen from Figure 11b that this grasping mode can also achieve the stable grasping. Then the slice with the thickness of 150 μm and the weight of 21.4 mg are picked up and placed by adopting the grasping mode where two fingers move simultaneously, as shown in Figure 11d.

## 6. Conclusions

A novel symmetric microgripper with two grasping modes has been designed, analyzed and tested in this paper. The structure of this microgripper can be divided into left part and right part, which are driven by two PZT actuators, respectively. In order to obtain desired output displacement, a three-stage amplification mechanism including two lever mechanism and a bridge-type mechanism is integrated into each part of the microgripper. Two grasping modes can be achieved by choosing one finger moving or two fingers moving simultaneously. Theoretical analysis and FEA are carried out to investigate the performance and optimize the parameters of the microgripper. Then, the prototype of the designed microgripper is fabricated by WEDM method and the experimental tests are carried out to verify the performance of the microgripper. The open-loop tests results show that the displacement amplification is 10.41 for each part and the natural frequency is 746.56 Hz. When the input voltage applied on the PZT actuators are 100 V, the grasping force applied on the copper wire with the diameter of 0.5 mm can reach 366.7 mN. PID position controller is adopted to compensate the hysteresis nonlinearity of the PZT actuated microgripper. The close-loop tests results indicate the motion resolution is 0.2 μm and the settling time is 39 ms for the step response. Finally, experiments are conducted to evaluate the grasping stability of the microgripper by operating the wires with different diameters and the slices with different thickness.

## Figures and Tables

**Table 1:** The features of different kind of microgrippers.

Types of the Microgripper	Advantages	Drawbacks
Electrothermal microgripper	Compact structure, large displacement and force	high operation temperature, nonlinear movement and low sensitivity
Electrostatic microgripper	Compact structure	Small grasping force
PZT-actuated microgripper	high force output to weight ratio, fast response and zero backlash	hysteresis nonlinearity

**Table 2:** The comparison with the existing microgrippers.

Literature	Number of Movable Fingers	Amplification Ratio	Stroke (μm)	Number of PZT Actuators
[15]	2	15.5	134	1
[17]	2	23.2	500	1
[18]	2	2.85	100	1
[19]	2	18.75	110	1
[22]	1	-	220	1
This work	1/2	20.82	236.68	2

**Table 3:** The material parameters of Al-7075.

Parameter	Density	Modulus of Elasticity	Yield Strength	Poisson’s Ratio
Value	2.81 × 10^3^ kg/m^3^	71.7 GPa	503 MPa	0.33
